# Prognostic Value of TRPM7 Expression and Factor XIIIa-Expressing Tumor-Associated Macrophages in Gastric Cancer

**DOI:** 10.1155/2021/7249726

**Published:** 2021-12-13

**Authors:** Muhammet Calik, Ilknur Calik, Gokhan Artas, Ibrahim Hanifi Ozercan

**Affiliations:** Department of Pathology, Faculty of Medicine, Fırat University, 23000 Elazığ, Turkey

## Abstract

**Purpose:**

TRPM7 is known to play a key role in tumor progression by regulating cellular proliferation, migration, and invasion in various cancer cell lines. However, there are no comprehensive clinical studies about the effect of TRPM7 expression on gastric cancer (GC) prognosis. In this study, it was aimed at investigating the effect of TRPM7 expression on prognosis in GC patients. Additionally, for the first time, it was investigated whether the density of Factor XIIIa-expressing tumor-associated macrophages (TAMs) in GC has an effect on the biological behaviour of the tumor.

**Methods:**

TRPM7 expression and Factor XIIIa-expressing TAM density were immunohistochemically evaluated in paraffin-embedded tumor tissues of 204 GC patients undergoing surgery at a single institution.

**Results:**

Tumor size was clearly higher in cases with high TRPM7 expression than those with low expression (*p* < 0.001, Mann-Whitney *U*). TRPM7 overexpression was closely related to high depth of tumor invasion (*p* < 0.001, ANOVA), increased lymph node metastasis (*p* < 0.001, ANOVA), and high distant metastasis rate (*p* < 0.001, Mann-Whitney *U*). These findings exposed that high TRPM7 expression is effective in the progression and aggressiveness of GC. In addition, while high CD8^+^ TIL density affects the prognosis positively, it was determined that high Factor XIIIa^+^ TAM density negatively affects the prognosis of patients with GC. Furthermore, multivariate analyses revealed TRPM7 overexpression was independently related with short overall (HR 9.64, 95% CI 5.74–16.19, *p* < 0.001) and disease-free survival (HR 5.67, 95% CI 3.61-8.92, *p* < 0.001) in GC patients.

**Conclusions:**

Our data suggest that high TRPM7 expression is closely related to progressive tumor behaviour in GC and independently negatively affects survival in patients. In addition, it was determined that a high density of Factor XIIIa^+^ TAMs negatively affects the prognosis of patients with GC.

## 1. Introduction

Gastric cancer (GC), the fifth most common cancer worldwide and the third leading cause of cancer-related death, remains a major public health problem [1–3]. Aggressive biological features of GC including high incidence, early relapse, and distant metastasis are responsible for poor prognosis and shorter 5-year follow-up times [4]. Therefore, more effective and reliable biomarkers are needed in terms of predictability of prognosis and development of new targeted treatment methods in GC.

Evidence revealing in recent years indicates that ion channels both regulate ion levels in cells, membrane potential, and intercellular signalling and also play significant roles in cell proliferation, migration, apoptosis, and differentiation [5]. The transient receptor potential (TRP) channels, first identified in 1969 by Cosens and Manning in Drosophila, are a family of non-voltage-dependent calcium-permeable ion channels [6, 7]. The TRP superfamily, which presently has 28 members, is divided into six subgroups as TRPA (ankyrin), TRPC (canonical), TRPM (melastatin), TRPML (mucolipin), TRPP (polycystine), and TRPV (Vanilloid) [7]. TRPM7 is a featured member of the TRPM family and has managed to attract the attention of cancer researchers in recent years [7]. Preliminary studies have demonstrated that in addition to its physiological functions, TRPM7 may play a key role in cancer by regulating cellular proliferation, migration, and invasion in cancer cell lines [8, 9]. Kim et al. suggested TRPM7 is overexpressed in several GC cell lines, including AGS which is one of the most common human gastric adenocarcinoma cell lines [10]. Additionally, in some studies using a variety of pharmacological agents, TRPM7 blockade has been shown to cause a marked decrease in cell proliferation and an increase in apoptosis. These findings implicated that TRPM7 could be a promising molecule for targeted therapies in GC [7]. In addition to GC, TRPM7 overexpression also has been shown in pancreatic adenocarcinoma, bladder cancer, and breast cancer cells [9, 11, 12]. Studies have determined that upregulation of TRPM7 is necessary for the proliferation of tumor cells. TRPM7 channels are permeable to both Ca^2+^ and Mg^2+^ ions. Since Ca^2+^ is an essential molecule for cell cycle and proliferation, regulation of Ca^2+^ influx is considered one of the mechanisms by which TRPM7 provides cell viability [9]. According to Jiang et al., TRPM7-mediated Ca^2+^ influx has played a substantial role in human head and neck carcinoma cell proliferation [13]. There are various in vitro studies on the importance of TRPM7 expression in many malignant tumor cell lines. However, clinical studies with large populations are needed to investigate the effect of TRPM7 expression in tumor tissues on the prognosis of patients with GC. Following these studies, TRPM7 may be considered as one of the targeted treatment options for GC.

The relationship between cancer and inflammation has been a topic of interest for years. There are studies demonstrating reliable evidence that inflammation and tumor microenvironment (TME) play important roles in the development and metastasis of many malignant neoplasms, including GC [14, 15]. The two main components of TME are tumor-infiltrating lymphocytes (TILs) and tumor-associated macrophages (TAMs). TILs and TAMs have been shown to have prognostic value in previous studies [14]. Macrophages have the ability to change their functional properties in response to changes in environmental conditions. When macrophages contact chemical mediators such as gamma interferon (IFN-*γ*) and lipopolysaccharides (LPS) in their microenvironment, they transform into M1 macrophages with antitumor activity. On the other hand, when exposed to mediators such as IL-4 and IL-13, they evolve into M2 macrophages that support tumor growth. TAMs in the TME mainly show mostly M2 macrophage phenotypic characteristics [16]. In previous studies, immune markers such as CD163, CD204, and CD206 were used in the identification of M2 macrophages [16, 17]. However, especially in M2 macrophages, Factor XIIIa expression has also been demonstrated [8, 18]. Recently, studies in various types of cancer such as lung, ovarian, and breast cancer have shown that high numbers of M2 macrophages in the TME are associated with poor prognosis [19–21]. In particular, it has been stated that macrophages expressing Factor XIIIa play an important role in invasion and metastasis in squamous cell cancer of the lung, which is associated with poor prognosis [19].

In this study, it was aimed at investigating the effect of TRPM7 expression on prognosis in gastric cancer patients. In our series of 204 GC cases, relationship between classical pathological prognostic parameters, the reliability of which has been demonstrated by many primary studies, and survival was examined. In addition, probably for the first time, it was investigated whether the density of Factor XIIIa-expressing macrophages in TME of GC has an effect on the biological behaviour of the tumor.

## 2. Materials and Methods

### 2.1. Patients and Basic Clinicopathological Features

This study population consisted of 204 patients who underwent surgery for GC in Fırat University Faculty of Medicine between 2009 and 2016. The cases who received any additional treatment, such as adjuvant or neoadjuvant chemotherapy, other than surgery were excluded from the sample. Clinical, radiological, and laboratory information of patients was obtained from the electronic archive centre of the hospital. Pathological samples of the patients were reevaluated by two pathologists in terms of tumor site, Borrmann type, histopathological tumor type, histological grade, Goseki grade, lymphovascular invasion, perineural invasion, TILs, depth of invasion (pT), lymph node metastasis (pN), and TNM stage. Histological tumor types were specified according to the criteria of the World Health Organisation (WHO) classification system and Lauren's classification. The TNM stages of the cases were determined according to the American Joint Committee on Cancer (AJCC), 7th edition. Survival data of the patients were collected from hospital medical data-processing records. The follow-up period was determined as 5 years. Overall survival (OS) and disease-free survival (DFS) times were determined as the interval between the dates of surgery and death or recurrence. The present study was approved by the Fırat University Ethical Committee (Date: 14 January 2021, Approval No: 2021/01-06).

### 2.2. Immunohistochemical Staining and Scoring Methods

Immunohistochemical staining was completed successfully using the indirect immunoperoxidase technique on formalin-fixed, paraffin-embedded tissues. In the staining process, below antibodies were used: anti-TRPM7 (bs-9044R, BIOSS antibodies, Woburn, MA, USA), anti-Factor XIIIa (MAD-000714QD-7 (EP292), Master Diagnostica, 41020-Sevilla, Spain), anti-CD4 (SP35, Ventana, Arizona, USA), and anti-CD8 (SP57, Ventana, Arizona, USA). Paraffinized slides obtained from pathology blocks containing GC tissues were automatically stained with the Ventana BenchMark Ultra coater and ultraView Universal DAB Kit (Ventana, Tucson, AZ-85755, USA) in accordance with the manufacturer's procedures that do not allow external intervention. The density and distribution of TRPM7 expression in tumor cells were evaluated immunohistochemically, ignorant of clinicopathological data. In addition, the intensity of TILs and TAMs was evaluated by CD4, CD8, and Factor XIIIa staining.

CD4^+^, CD8^+^ TIL density, and Factor XIIIa^+^ TAM intensity were assessed holding to earlier studies [14, 22]. Briefly, five fields with the intense infiltration of inflammation were selected from each staining slide and the percentages of CD4^+^, CD8^+^ TILs, and Factor XIIIa^+^ TAMs were evaluated. The mean of these five areas was put to use as the density. Firstly, a semiquantitative staining score was specified according to the following features: 1 (<1% cells), 2 (1-10% cells), 3 (11-33% cells), 4 (34-66% cells), and 5 (67-100% cells). While evaluating positivity, partial or complete positivity in the cytoplasm and/or plasma membrane of inflammatory cells was considered. Secondly, intensity of immunoreactivity was scored as follows: 0 (absent), 1+ (slight), 2+ (moderate), and 3+ (intense). Finally, TIL and TAM IHC scores were calculated by adding the mean staining scores and the intensity for each slide (ranging from 1 to 8). The mean ± standard deviation (SD) value of the IHC score (3.41 ± 1.54, 3.17 ± 1.85, and 3.59 ± 1.73, respectively) was used as a cut-off criterion to group the patients as low and high intensity in terms of CD4, CD8, and Factor XIIIa.

The expression of TRPM7 in GC was assessed using the IHC scoring method confirmed in previous studies [23, 24]. During evaluation process, cytoplasmic and membranous staining in tumor cells were taken into consideration. IHC score was obtained multiplying the staining intensity (0: no staining, 1: weak staining (light yellow), 2: moderate staining (yellow brown), and 3: strong staining (brown)) by the percentage of positively stained cells (0: <5%; 1: 5% −20%; 2: 21% −50%; and 3: >50%.). IHC score ranged from 0 to 9. The mean value of the IHC score (3.71 ± 3.16) was accepted as the cut-off criterion. The cases with IHC score below this value were considered as a low-TRPM7 expression group and those above were characterized as a high-TRPM7 expression or overexpression group.

### 2.3. Survival and Statistical Analysis

In this study, SPSS version 26 software (IBM Corporation, Armonk, NY, USA) was used for statistical analysis of data. Obtained outcomes were expressed as percentages, medians, interquartile range (IQR), means, and SD. Normality was checked by the Shapiro-Wilk test. Also, the kurtosis and skewness values between −0.5 and +1.5 were accepted to demonstrate normality. Independent sample *t*-test and ANOVA were used to determine the differences between groups with normal distribution. While performing multiple comparisons in ANOVA, Dunnett's T3 and Bonferroni tests were used according to the equality of variances after Bonferroni correction at the significance level. On the other hand, the Mann-Whitney *U* test was preferred to determine the differences between groups without normal distribution. Survival analysis was performed using the Kaplan-Meier method. The log-rank test was used to compare the survival rate for each variable. In addition, a multiple Cox proportional hazard regression model (using the “enter” method) was conducted to examine the effects of TRPM7 expression, TILs, and Factor XIIIa-expressing TAM density on overall survival (OS) and disease-free survival (DFS) in patients with CRC. In order to investigate the effects of independent variables (confounding), basic classical prognostic parameters, which were shown to have an effect on the survival of GC patients in previous years, were included in the model. Among these parameters, those with a *p* value less than 0.05 in univariate Cox regression analysis such as tumor site, lymphovascular invasion, histological grade, pT, pN, and TNM stage were selected. Then, age and gender data, which are epidemiologically important parameters, were also added to the model to reduce bias. For all statistical data, a *p* value less than 0.05 was considered statistically significant.

## 3. Results

### 3.1. Some of the Key Clinical and Pathological Parameters Were Closely Related to Survival

126 (61.8%) of the patients included in this study were male and 78 (38.2%) were female. The age of the patients ranged from 25 to 85, with a mean age of 64.48 ± 13.34 years. Most of the tumors were settled in the distal (*n* = 76, 37.3%) and proximal (*n* = 56, 27.2%) site of the stomach. According to the classification of Lauren, 122 (59.8%) of the cases were intestinal, 68 (33.3%) diffuse, and 14 (6.9%) indeterminate histological types. Most of the patients in this study had advanced TNM stage, of whom 76 (%37.3) were stage III and 60 (%29.4) were stage IV. During the five-year follow-up, 164 deaths occurred. The five-year survival rate of patients in this series was 19.6%. OS and DFS times were determined as 37.40 ± 14.06 and 30.15 ± 15.11 months, respectively. The earliest recurrence was seen in the 9th month from onset.

In this series, there was no significant relationship between the classical prognostic parameters such as gender, age, tumor size, Borrmann classification, histopathological tumor type, Goseki grade and perineural invasion, and the survival of patients with GC (Table 1). Likewise, these parameters had no effect on DFS. In contrast, there was a highly significant correlation between tumor site, histological grade, lymphovascular invasion, pT, pN, distant metastasis, and TNM stage, and both OS and DFS, according to univariate analyses. The 5-year survival rates of patients with gastroesophageal tumor (33.65 ± 13.81 months) were significantly lower than those with middle and distal localization (37.79 ± 12.34 and 44.55 ± 14.09 months, respectively). The worst survival times were seen in patients with diffuse gastric involvement (31.52 ± 12.89 months). In univariate analyses, it was determined that OS and DFS rates decreased significantly as histological grade, depth of tumor invasion, and lymph node metastasis increased. In addition, while 5-year OS was 58.65 ± 3.84 months in TNM stage I patients, it was reduced to 23.56 ± 6.23 months in those with stage IV. When the data were examined in multivariate Cox regression analysis, it was seen that only the TNM stage could be used as independent prognostic indicators among classical prognostic parameters (Table 2). In addition, disease-free survival rates were significantly lower in patients with advanced TNM stage. According to our model, advanced clinical stage was found to be a very high-risk factor for early recurrence (HR 3.07, 95% CI 2.26-4.18, *p* < 0.001) (Table 3). These findings emphasized that advanced TNM stage is a strong and independent indicator of poor prognosis in patients with GC.

### 3.2. CD4^+^ and CD8^+^ TIL Density Was Closely Related to Prognosis in Patients with Gastric Cancer

**Figure 1:**
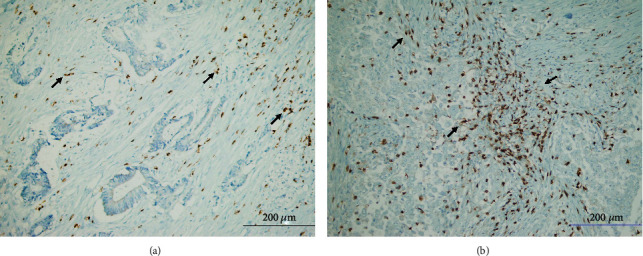
Representative TIL density in gastric cancer tissues: (a) low CD4^+^ TIL density (arrows) (CD4x200); (b) high CD4^+^ TIL density (arrows) (CD4x200).

**Figure 2:**
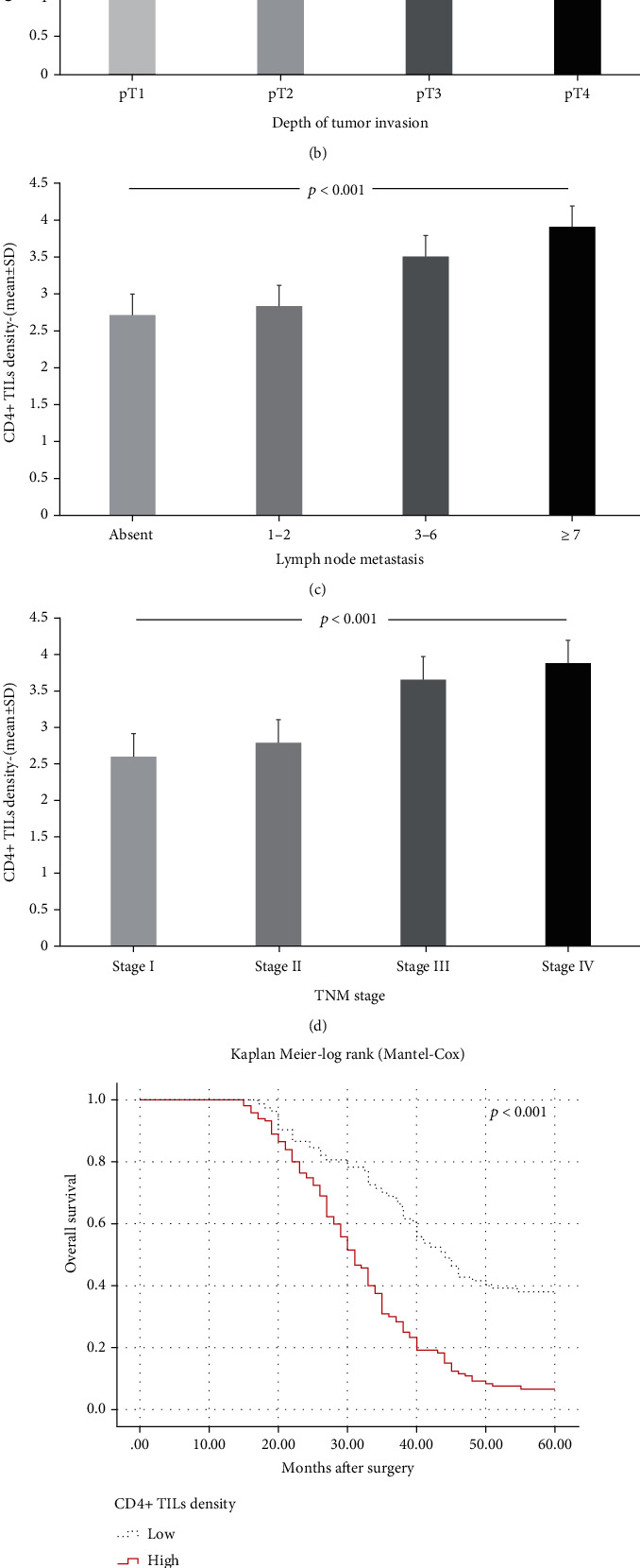
The differences of CD4^+^ TIL density according to classical prognostic parameters. (a) The density of CD4^+^ TILs was higher in patients with lymphovascular invasion (4.0, (range 7.0)) compared to those without (2.0, (range 5.0)) (*p* = 0.003, Mann-Whitney *U*). (b) As the depth of tumor invasion increased, the density of CD4^+^ TILs increased (pT1 = 2.58 ± 1.16, pT2 = 2.83 ± 1.46, pT3 = 3.52 ± 1.61, and pT4 = 3.62 ± 1.40) (*p* = 0.035, ANOVA). (c) There was a positive correlation between lymph node metastasis and high CD4^+^ TIL density (*p* < 0.001, ANOVA). (d) Similarly, TNM stage IV patients had a more intense infiltration of CD4^+^ TILs (3.88 ± 1.51) compared to TNM stage I patients (2.60 ± 1.18) (*p* < 0.001, ANOVA). In the post hoc test (Bonferroni), it was determined that there is a statistically significant difference in terms of CD4^+^ TIL density especially between stage IV patients and early stage (stage I-II) patients (*p* = 0.005 and *p* = 0.001, respectively). According to Kaplan-Meier analysis, the high CD4^+^ TIL density was significantly associated with both (e) overall (*p* < 0.001) and (f) disease-free survival (*p* < 0.001, log rank).

CD4^+^ TIL density was low in 84 (41.2%) cases and high in 120 (58.8%) cases in this series (Figure 1). There was no correlation between CD4^+^ TIL density and tumor site (*p* = 0.101, ANOVA), histological type (*p* = 0.357, ANOVA), histological grade (*p* = 0.185, ANOVA), and perineural invasion (*p* = 0.762, Mann-Whitney *U* test). On the other hand, there was a positive correlation between the density of CD4^+^ TILs and parameters such as pT, lymphovascular invasion, pN, and TNM stage, which are important indicators of tumor progression. CD4^+^ TIL density was found to be high in patients with lymphovascular invasion and advanced stages of pT, pN, and TNM (Figures 2(a)–2(d)). Moreover, supporting these findings, OS and DFS times were significantly shorter in patients with high CD4^+^ TILs in Kaplan-Meier analysis (Figures 2(d) and 2(e)). However, there was no effect of CD4^+^ TIL density on survival in multivariate analysis (Tables 2 and 3).

**Figure 3:**
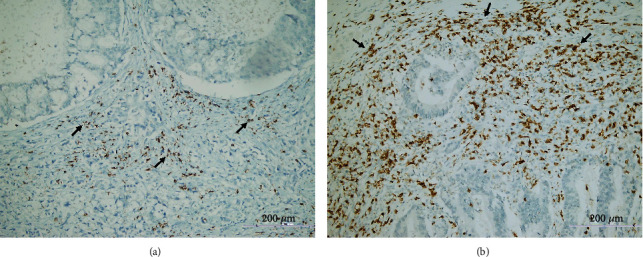
Representative TIL density in gastric cancer tissues: (a) low CD8^+^ TIL density (arrows) (CD8x200); (b) high CD8^+^ TIL density (arrows) (CD8x200).

**Figure 4:**
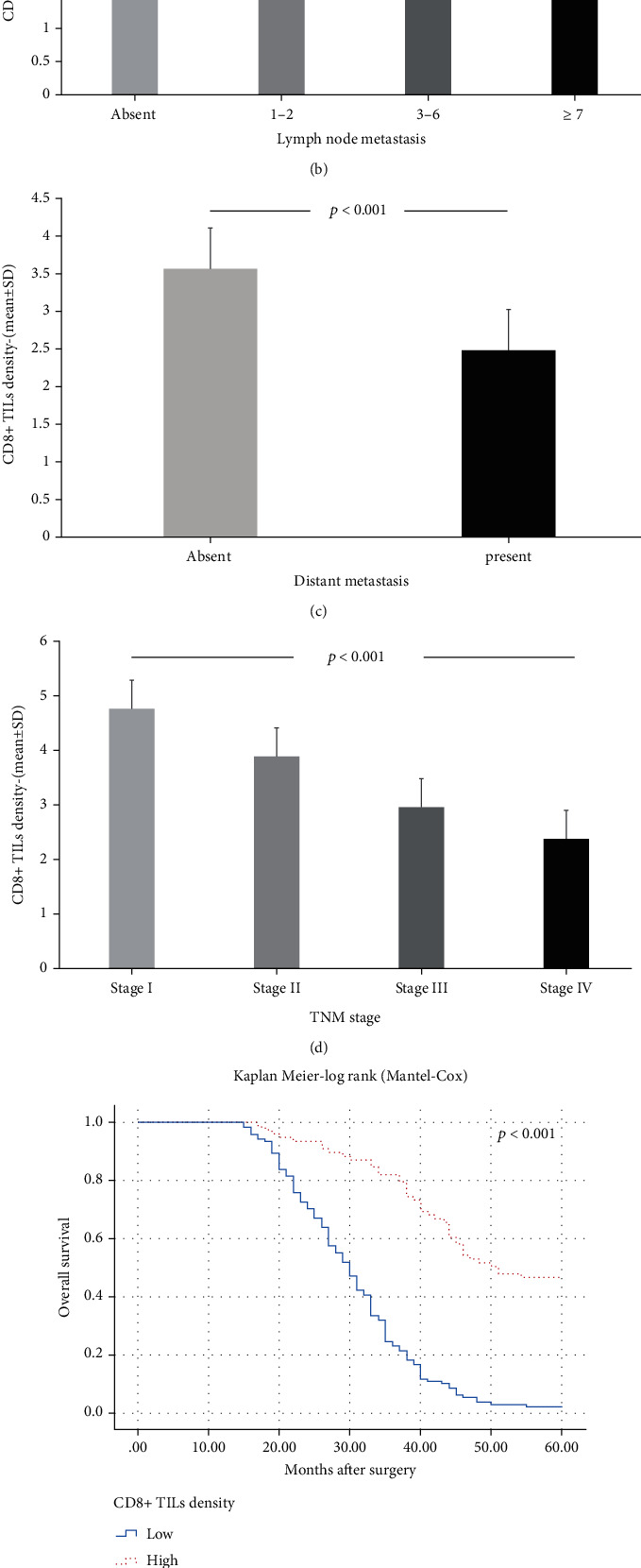
There was a negative correlation between high CD8^+^ TIL density and basic prognostic parameters including depth of tumor invasion, lymph node metastasis, distant metastasis, and TNM stage. As the density of CD8^+^ TILs increased, (a) the depth of tumor invasion (*p* < 0.001, ANOVA), (b) lymph node metastasis (*p* < 0.001, ANOVA), (c) distant metastasis rates (*p* < 0.001, ANOVA), and (d) TNM stage (*p* < 0.001, ANOVA) decreased. According to Kaplan-Meier survival analysis, high CD8^+^ TIL density was significantly associated with longer (e) overall (*p* < 0.001, log rank) and (f) disease-free survival (*p* < 0.001, log rank).

When the immunohistochemical staining was evaluated, CD8^+^ TIL density was found to be low in 125 (61.3%) cases and high in 79 (38.7%) cases in this series (Figure 3). Contrary to the density of CD4^+^ TILs, high CD8^+^ TIL density was seen to be an indicator of a good prognosis. There was a negative correlation between high CD8^+^ TIL density and key prognostic parameters such as pT, pN, distant metastasis, and TNM stage. The pT, pN, distant metastasis rate, and TNM stages of the patients with high CD8^+^ TIL density were significantly lower than those with low CD8^+^ TIL density (Figures 4(a)–4(d)). According to Kaplan-Meier survival analysis, high CD8^+^ TIL density had a positive effect on survival. Both OS and DFS times of patients with high CD8^+^ TIL density were significantly higher than those with low CD8^+^ TIL density (Figures 4(e) and 4(f)). Moreover, high CD8^+^ TIL density appeared to be associated with longer survival times in multivariate analysis (Tables 2 and 3). These findings revealed that the density of CD8^+^ TILs is an independent indicator of a good prognosis for patients with GC.

### 3.3. Factor XIIIa-Expressing TAMs Negatively Affected Survival in Univariate Analyses

**Figure 5:**
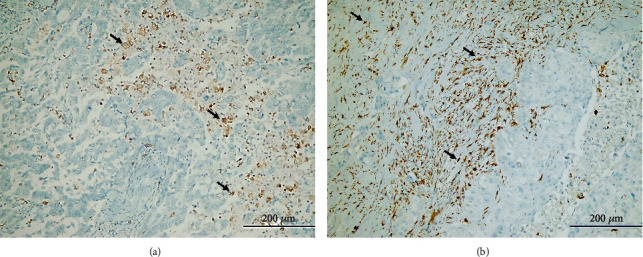
Factor XIIIa-expressing tumor-associated macrophages in tumor microenvironment of gastric cancer tissues: (a) low Factor XIIIa^+^ tumor-associated macrophage density (arrows) (FactorXIIIax200); (b) high tumor-associated macrophage density (arrows) (FactorXIIIax200).

**Figure 6:**
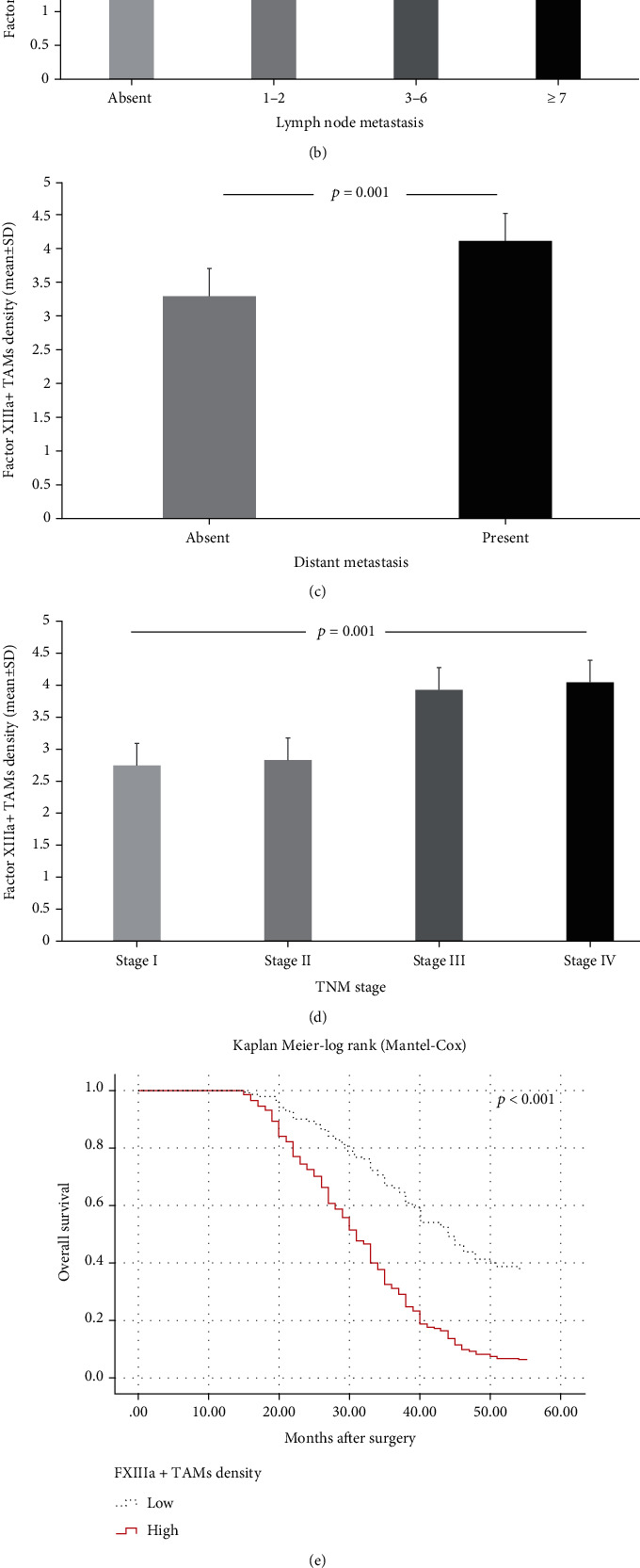
Relationship between Factor XIIIa-expressing tumor-associated macrophage density and classical prognostic parameters and prognosis. (a) Tumor size was larger (5.0 cm (2.5, interquartile range)) in a group with high Factor XIIIa-expressing tumor-associated macrophage density compared to those with low one (4.0 cm (3.0, interquartile range)). As the density of Factor XIIIa-expressing tumor-associated macrophages increased, (b) lymph node metastasis (*p* < 0.001, ANOVA), (c) distant metastasis (*p* = 0.001, ANOVA), and (d) TNM stage (*p* = 0.001, ANOVA) also increased. In Kaplan-Meier analysis, high Factor XIIIa-expressing tumor-associated macrophage density was significantly associated with both (e) shorter overall (*p* < 0.001, log rank) and (f) disease-free survival times (*p* < 0.001, log rank).

In this study, immunohistochemical staining was applied to 204 gastric cancer tissues to evaluate the effect of Factor XIIIa-expressing TAM density on tumor progression and prognosis of GC patients. Patients in the study population were divided into two groups according to the IHC score as low and high Factor XIIIa^+^ TAM density. 86 (42.2%) of the cases had low Factor XIIIa^+^ TAM intensity, and 118 (57.8%) had high Factor XIIIa^+^ TAM density (Figure 5). There was a positive correlation between tumor size and Factor XIIIa^+^ TAMs (Figure 6(a)). Significantly higher density of Factor XIIIa^+^ TAMs (4.0, (3.0, interquartile range)) was detected in patients with large tumor size compared to those with small size (3.0, (2.0, interquartile range)) (*p* = 0.007, Mann-Whitney *U*). In addition, it was observed that the tumor invaded deeper in patients with a high density of Factor XIIIa^+^ TAMs (*p* = 0.024, ANOVA). When viewed in terms of other parameters, there was no statistical correlation between Factor XIIIa^+^ TAM density and tumor site (*p* = 0.069, ANOVA), histological tumor type (*p* = 0.147, ANOVA), and histological grade (*p* = 0.112, ANOVA). However, the high density of Factor XIIIa^+^ TAMs was closely related to increased pN, high distant metastasis rates, and advanced TNM stage (Figures 6(b)–6(d)). Whether factor XIIIa^+^ TAM density has an effect on survival was investigated by the Kaplan-Meier method. According to our data, high Factor XIIIa^+^ TAMs had a significant negative effect on OS and DFS (Figures 6(e) and 6(f)). Further, in univariate Cox regression analysis, the risk of death (HR 2.81, 95% CI 2.01-3.94, *p* < 0.001) and recurrence (HR 2.65, 95% CI 1.94-3.62, *p* < 0.001) was significantly higher in patients with a high density of Factor XIIIa-expressing TAMs than those with low density. However, no significant relationship was determined between Factor XIIIa^+^ TAMs and survival in multivariate analyses (Table 2).

### 3.4. High TRPM7 Expression Arisen as an Independent Indicator of Poor Prognosis in Gastric Cancer

**Figure 7:**
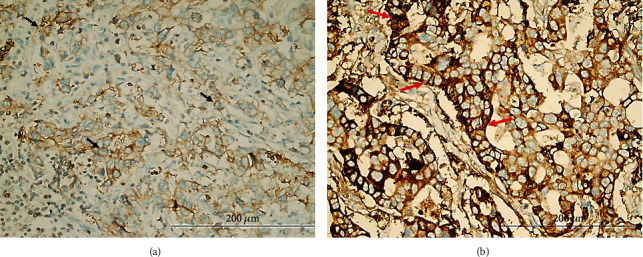
Representative figures of TRPM7 expression in human gastric cancer specimens: (a) low cytoplasmic TRPM7 expression in tumor cells (black arrows) (TRPM7x400); (b) high expression of TRPM7 in tumor cells (red arrows) (TRPM7x400).

**Figure 8:**
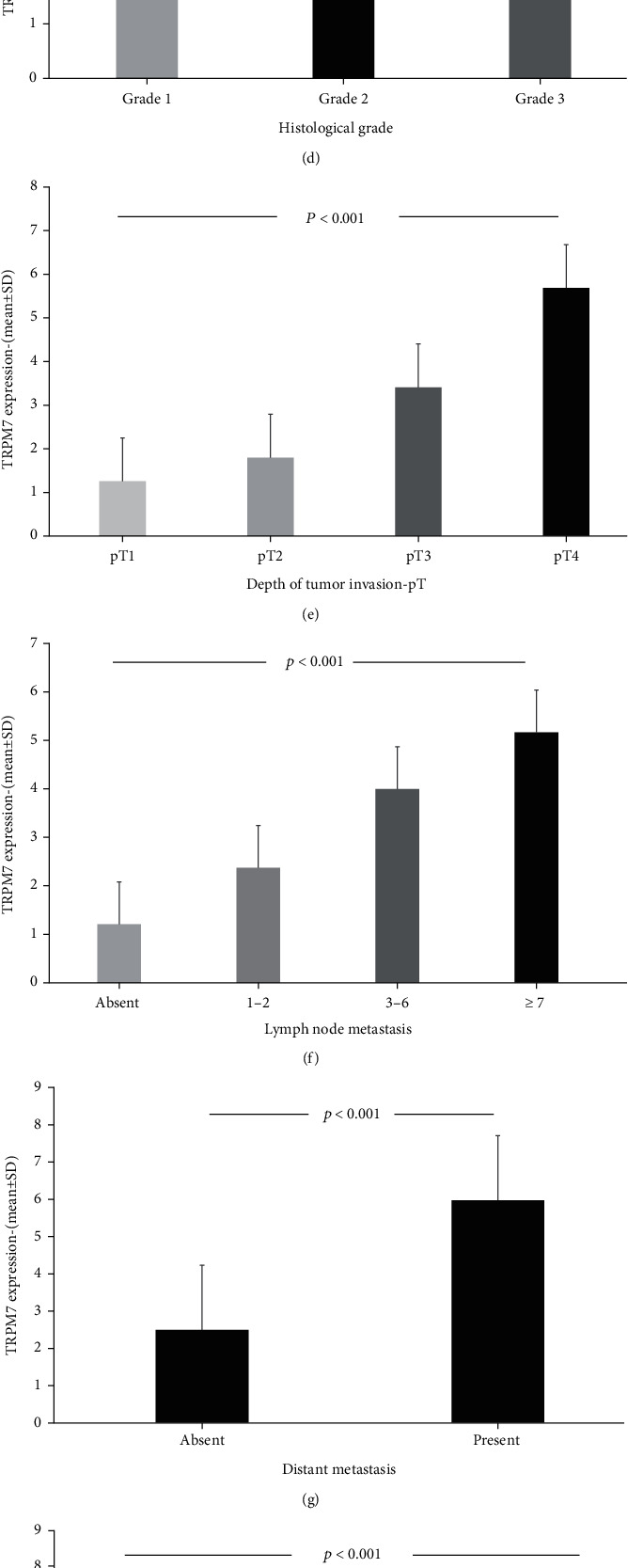
Relationship between TRPM7 expression and classical clinicopathological prognostic parameters and survival. (a) Tumor size was significantly larger in cases with high TRPM7 expression (3.0 (6.5, interquartile range)) compared to those with low expression (1.0 (1.0, interquartile range)) (*p* < 0.001, Mann-Whitney *U*). (b) TRPM7 expression was higher in cases with proximal site and diffuse involvement (*p* < 0.001, ANOVA). (c) The highest TRPM7 expression was seen in the indeterminate tumor type group among histopathological tumor types, according to Lauren (*p* = 0.022, ANOVA). (d) TRPM7 expression was higher in cases with lymphovascular invasion (2.0 (5.75, interquartile range)) compared to those without (1.5 (5.0, interquartile range)) (*p* = 0.045, Mann-Whitney *U*). (e) TRPM7 expression increased with loss of differentiation in tumors. TRPM7 expression was significantly higher in poorly differentiated tumors (4.32 ± 3.22) compared to those with well differentiated (1.83 ± 2.23) (*p* = 0.005, ANOVA). Additionally, high expression of TRPM7 was positively correlated with (f) increased tumor invasion depth (*p* < 0.001, ANOVA), (g) lymph node metastasis (*p* < 0.001, ANOVA), (h) distant metastasis (*p* < 0.001, *t*-test), and advanced (i) TNM stage (*p* < 0.001, ANOVA). According to Kaplan-Meier survival analysis, (a) patients with high TRPM7 expression had a poor overall survival (*p* < 0.001, log rank). (b) The cases with TRPM7 expression had shorter disease-free survival rates than those with low expression (*p* < 0.001, log rank).

To evaluate the clinical significance of TRPM7 in the biological behaviour of GC, we investigated the TRPM7 expression profile in cancerous and noncancerous tissues belonging to the same patient by immunohistochemistry. Expectedly, TRPM7 was highly expressed in GC tissues compared to adjacent noncancerous ones. TRPM7 immunoreactivity was demonstrated in both the cytoplasm and the cytoplasmic membrane of tumoral cells. The staining obtained on the cytoplasmic membrane was slightly more intense compared to the cytoplasm. The cases were divided into two groups according to the IHC score as low (*n* = 127, 62.3%) and high (*n* = 77, 37.7%) TRPM7 expression groups (Figure 7). When the relationship between TRPM7 expression and clinicopathological features is examined, a significant correlation was determined between TRPM7 expression and classical prognostic parameters such as tumor size, histopathological type (Lauren classification), lymphovascular invasion, histological grade, pT, pN, distant metastasis, and TNM stage (Figures 8(a)–8(h)). However, there was no any correlation between TRPM7 expression and other prognostic parameters such as gender, age, Borrmann type, histological type (WHO), and perineural invasion. In addition, the relationship of TRPM7 with TME was investigated. Interestingly, there was a negative correlation between TRPM7 expression and the density of CD8^+^ TILs. However, a positive correlation was determined between TRPM7 expression and the density of CD4^+^ TILs, and Factor XIIIa^+^ TAMs. The patients with TRPM7 overexpression were found to have a higher density of Factor XIIIa^+^ TAMs (4.24 ± 1.59) compared to those with low expression (3.19 ± 1.69) (*p* < 0.001, *t*-test). So as to investigate comprehensively the prognostic significance of TRPM7 expression in patients with GC, Kaplan-Meier survival analysis and Cox regression analyses were performed. According to the log-rank test, cases with high TRPM7 expression were found to have a significantly worse prognosis compared to those with low TRPM7 expression. Patients with high TRPM7 expression had statistically significantly shorter OS and DFS times than those with low expression (Figures 8(i) and 8(j)). The multivariate analyses demonstrated high TRPM7 expression was independently related with poor OS (HR 8.70, 95% CI 5.20–14.54, *p* < 0.001) (Table 2) and DFS (HR 4.57, 95% CI 2.93-7.12, *p* < 0.001) (Table 3) in GC patients. According to our regression model, it was clearly revealed that TRPM7 expression, together with TNM stage and CD8^+^ TIL density, can be used as an independent predictor of prognosis in patients with GC.

## 4. Discussion

Ever since Hanahan and Weinberg described some of the features that a healthy cell must acquire in the process of transforming into a cancer cell about 20 years ago, these distinctive features of cancer have continued to evolve until today. However, its basic principles are still maintained [25, 26]. It is known that ion channels affect many cellular activities that are largely related to these properties of cancer [25]. Based on this perspective, it has been questioned whether cancer is oncochannelopathy or not lately [26]. To illustrate, it has been established that Ca^2+^ channels play important roles both in the control of cellular growth and proliferation and in the control of cell death [25]. In recent years, several studies reporting that TRPM7 plays an important role in carcinogenesis and claim that it can be considered as a potential target in the treatment of various cancers have attracted attention [9, 12]. As Ca^2+^ is an important regulator of cell cycle and proliferation, TRPM7, which is a special member of the TRP channels known to be permeable to Ca^2+^ and Mg^2+^, is thought to be highly important in terms of cancer cell biology [9]. There are studies related to the effects of TRPM7 expression on cellular proliferation and migration in various cancer cell lines [8, 9]. However, there are no comprehensive clinical studies on the effect of TRPM7 on the biological behaviour and prognosis of GC. The primary aim of the present study is to investigate the prognostic value of TRPM7 expression in GC patients.

TRPM7 appears to be necessary for the proliferation and cell migration of various cancer cells including breast cancer, GC, pancreatic cancer, bladder cancer, head and neck cancer, and ovarian cancer [9–13, 28]. It is reported that TRPM7 is overexpressed in human pulmonary carcinoma and pancreatic adenocarcinoma [29, 30]. In addition, the inhibition and downregulation of TRPM7 by specific chemical agents is known to inhibit the migration and invasion of breast cancer cells, while overexpression has been shown to support the proliferation and migration of lung cancer cells [12, 29]. According to Luanpitpong et al., blocking of TRPM7 has repressed cell motility in various non-small-cell lung carcinoma cell lines and patient-derived primary tumor cells. They also found that downregulation of TRPM7 reduced lung metastases, which is formed experimentally. Furthermore, according to their results, high TRPM7 channel expression has correlated with the low survival times of patients and high metastatic rates [29]. Chen et al. suggested that TRPM7 expression was higher in prostate carcinoma cells compared to prostatic hyperplasia. They stated that reduced TRPM7 repress cell migration and invasion in prostate cancer cell lines, while TRPM7 overexpression increased the migration of prostate cancer cells [31]. In the study of Kim et al., overexpression of TRPM7 has been identified in several gastric cancer cell lines including AGS which is one of the best-known human gastric adenocarcinoma cell lines [10]. Later, it is stated that transfection of AGS cells with TRPM7 small interfering RNA (siRNA) significantly reduced the expression of TRPM7. Moreover, they showed that Mg^2+^ is crucial for the growth and survival for AGS cells. In their study, it is clearly implicated blocking of TRPM7 channels by La^3+^ and 2-APB or repression of TRPM7 expression by siRNA inhibited the growth and survival of these cells, ultimately. In another study of Kim et al., the role of TRPM7 channels in Ginsenoside Rg3-inhibited AGS cells growth and survival has been elucidated. In their study, Ginsenoside Rg3 was determined to inhibit the growth and survival of AGS cells. Subsequently, suppression or complete blocking of TRPM7 expression was found to block Rg3-induced inhibition of cell growth and survival. Moreover, overexpression of TRPM7 channels in HEK 293 cells has been shown to increase the rate of Rg3-induced cell death. These findings suggest that Ginsenoside Rg3 inhibits the growth and survival of gastric cancer cells through the blocking of TRPM7 channel activity. Ultimately, based on these results, it has been claimed that TRPM7 expression may play an important role in survival in patients with gastric cancer. Beyond the above-mentioned experimental and cell line studies investigating the importance of TRPM7 expression in GC, it is obvious that clinical studies involving a large patient population are needed. In the present study, the effects of TRPM7 expression in tumoral tissues of 204 GC patients on tumor biology and its prognostic significance were investigated. In harmony with the findings obtained in previous studies that TRPM7 supports cell proliferation and tumor growth in various cancer cell lines, in our series, tumor size was significantly greater in patients with high TRPM7 expression than those with low expression. Also, in patients with high TRPM7 expression, the tumors were deeper invasive and high grade, which had a significant loss of differentiation. Taken together with previous studies, these findings support that upregulation of TRPM7 ion channels is associated with tumor progression and aggressive biological behaviour in GC.

Both regional lymph node and distant organ metastases are considered to be the most basic biological features of malignant tumors [28]. Former studies have demonstrated that the epithelial-mesenchymal transition (EMT) process is an early step of invasion and metastasis [28, 32]. Various features of cells change during EMT process, such as loss of intercellular adhesion, aggressive behaviour, and metastatic potential. The cells lose their epithelial phenotype and begin to acquire mesenchymal phenotypical characteristics [31]. In recent studies, it has been stated that calcium-related signalling through the PI3K/AKT pathway is very important for the EMT process in cancers [28, 33]. High intracellular calcium (i-Ca^2+^) levels are thought to stimulate the EMT process and metastasis by regulating the expression of molecules such as E-cadherin, N-cadherin, Vimentin, and *α*-smooth muscle actin (*α*-SMA) [28]. TRPM7 is one of the key molecules in the regulation of i-Ca^2+^ levels and has important roles in cancer development, progression, and metastatic cascades. Liu et al.'s study in ovarian carcinomas suggested that TRPM7 expression was negatively correlated with E-cadherin but positively with N-cadherin and Vimentin expression. Suppression of TRPM7 was determined to inhibit migration and invasion in SKOV3 and OVCAR3 cells, which are well-known ovarian cancer cell lines. Moreover, TRPM7 suppression was seen to reduce lung metastasis of SKOV3 tumor cells and extended survival of mice with ovarian cancer. According to their results, TRPM7 suppression inhibits EMT process and ovarian cancer metastasis through reducing calcium-related PI3K/AKT activation [28]. Their former study has revealed that TRPM7 overexpression is apparently seen in ovarian cancer tissues and cells, especially in metastatic ovarian cancer tissues. Additionally, TRPM7 upregulation was positively correlated with lymph node involvement and poor prognosis in patients with ovarian cancer [34]. Su et al. reported that the downregulation of TRPM7 expression in colorectal cancer (CRC) cells reversed the EMT process with a decrease in N-cadherin and an increase in E-cadherin [35]. Decreased TRPM7 expression has been shown to inhibit CRC cell proliferation, migration, and invasion in vitro. In addition, they observed apparently upregulation of TRPM7 expression in CRC tissues. CRC patients with TRPM7 overexpression had deeper tumor infiltration, positive lymph node metastasis, distant metastasis, and advanced clinical stage compared to those with low expression [35]. In our study, we obtained results consistent with previous research. One of the important findings emerging in the present study was the positive correlation between TRPM7 overexpression and metastasis. In patients with high TRPM7 expression, both the number of metastatic lymph nodes and the rates of distant metastasis were statistically significantly higher than those with low expression. We have also determined that TRPM7 overexpression has a negative effect on the survival of patients with GC. Furthermore, according to the results of multivariate analysis, it was revealed that upregulation of TRPM7 could independently be an indicator of poor prognosis in GC. When previous studies and our findings are evaluated together, it can be implicated that TRPM7 supports the EMT process by changing i-Ca^2+^ levels through the PI3K/AKT pathway and is associated with aggressive biological behaviour, metastasis, and poor survival in various cancers including GC. In the present study, it was clearly shown that there is a close relationship between high TRPM7 expression and aggressive biological features such as invasion, metastasis, and shorter survival times. However, further studies at the molecular level are needed to understand the mechanisms of these effects of TRPM7 expression in GC, and perhaps to reveal new targeted treatment options.

TME consists of various cellular or extracellular components such as lymphocytes, macrophages, endothelial cells, perivascular cells, fibroblasts, neurons, adipocytes, and extracellular matrix (ECM) and is thought to play an important role in invasion and metastasis of cancers [15]. TILs and TAMs are considered to be the most important elements in TME in terms of tumor biology, and their prognostic value has been demonstrated in many cancers [14, 15, 20]. However, previous studies have shown incompatible results regarding the relationship between TIL density and survival of patients with GC [36, 37]. For instance, Fukuda et al. reported that there was no significant variation in survival between patients with low or high TIL density [36]. Contrarily, Liu et al. demonstrated that patients with high CD8^+^ TILs have a longer 5-year survival than those with low CD8^+^ TILs [37]. Additionally, they claimed CD8^+^ TIL density can be used as an independent prognostic parameter, which is coincided with our results. In the present study, the utility of high CD8^+^ TIL density together with the TNM stage as independent prognostic parameters was revealed. However, the high density of CD4^+^ TILs negatively affected the prognosis of patients with GC. There may be several reasons for these conflicting results. First, there may be differences between immunohistochemistry and other methods used to determine the density of CD8^+^ TILs. Second, partial subjectivity due to the nature of the semiquantitative scoring systems used in TIL staining intensity assessment may adversely affect the results. Third, cross-reactions that occur during immunohistochemical staining may cause mismatches in the positive cell ratio. Additionally, the function of CD8^+^ TILs may differ depending on other immune cells in TME, and recent results have shown that TAMs play an important role in cancer aggressiveness. TAMs are an especial group of inflammatory cells that are key regulators of TME. They are thought to play critical roles in tumor progression, tumor growth, and metastasis [15]. TAMs are divided into two groups, classically activated macrophages (M1) and alternatively activated macrophages (M2) [14]. Most of macrophages in TME are inflammatory cells exhibiting M2 phenotypic characteristics and possessing angiogenic activity, promoting tumor growth and metastasis [15, 16]. As well as supporting angiogenesis, TAMs have been shown to have various protumor effects such as secretion of growth factors and matrix proteases and suppression of adaptive immunity. The antibodies such as CD163, CD204, and CD206 usually have been used to identify M2 macrophages, immunohistochemically [16, 17]. However, Factor XIIIa-expressing M2 macrophages have been also demonstrated recently in accordance with this purpose in some malignant tumors including lung cancer [19]. High intensity of TAMs is associated with a poor prognosis in many types of cancer [14–17, 19–21]. However, there is no consensus regarding the effect of TAMs on prognosis and clinicopathological features of GC. Many studies on this topic have shown that high TAMs density is associated with aggressive tumor biology and poor prognosis [14–16]. Contrary to these, some studies have different opinions [38]. Ohno et al. revealed that high TAM aggregation within the tumor nest has a good effect. They stated that TAMs in the tumor nest increased the frequency of apoptosis of tumor cells. According to their results, the 5-year disease-free survival rate in the group with the high density of TAMs was significantly higher than those with the low density [38]. Contrary, Lu et al. found out a negative correlation between TAMs and the prognosis of patients with GC [12]. They stated that when compared with CD68^+^ TAM negative patients, DFS rates were significantly lower in CD68^+^ TAM positive ones [14]. In a comprehensive meta-analysis, Wang et al. stated that TAMs participated in the progression of GC in vitro and high TAM infiltration were significantly positively correlated with TNM stage in GC patients [39]. They found that the expression of a gene called Kindlin-2 is upregulated at both mRNA and protein levels in GC cells cocultured with TAMs. Later, they showed that Kindlin-2 and increased TAMs were associated with a higher invasion rate. Additionally, it was shown that TAMs secreted TGF*β*2, increasing the expression of Kindlin-2 via the transcription factor NF-кB. Therefore, they stated that TAMs provided the progression and metastasis of GC through the TGF*β*2/NF-*κ*B/Kindlin-2 axis [39]. In a similar study, the results of Guo et al. revealed that TAMs promote the invasion and migration of GC cells in vitro [15]. They demonstrated that TAMs stimulate EMT in GC cells via the forkhead box Q1 (FOXQ1) and FOXQ1 is necessary for metastasis in GC cells. Moreover, they determined FOXQ1 silencing inhibits the TAM-induced EMT and metastasis of GC cells. They also stated that high CD68 expression in tissues of patients with GC correlated positively with FOXQ1 expression. Ultimately, their data provided evidence that TAMs stimulate EMT, invasion, and migration of GC cells through FOXQ1. Therefore, they claimed that the TAM/FOXQ1 axis could represent a new target for GC cells [15]. In a recent study, Porrello et al. demonstrated, with immunocompetent metastasis models, that tumor cell-derived CCL2-mediated inflammatory monocytes (IMs) are required and adequate for metastasis of lung squamous carcinomas (LUSC) [19]. In particular, they indicated that IMs extremely express Factor XIIIA, which promotes fibrin crosslinking to form a scaffold for LUSC cell invasion and metastases [19]. In the present study, we used the Factor XIIIa antibody to investigate the effect of M2 macrophage density on the prognosis of patients with GC. Our data was highly consistent with previous studies and supported its findings. High Factor XIIIa^+^ TAM density in GC was closely associated with larger tumor size and deeper tumor invasion, indicating its efficacy in tumor progression. Additionally, we obtained findings indicating that a high intensity of Factor XIIIa^+^ TAMs in GC supports not only local aggression but also distant metastasis. As a result of its effects on aggressive tumor biology, high density of Factor XIIIa^+^ TAMs in GC was significantly correlated with shorter OS and DFS times. However, unlike CD8^+^ TILs and TRPM7, we determined that high factor XIIIa^+^ TAM density cannot be used as an independent prognostic parameter.

Recent studies have revealed that TRPM7 also has effects on the functions of macrophages and plays a regulatory role for the homeostasis of the immune system [40–42]. Li et al. stated that TRPM7 blockade is an essential factor in the transformation of macrophages into M2 phenotype [40]. On the contrary, Schilling et al. suggested that TRPM7 channel activity is required for the macrophage polarization event where bone marrow-derived macrophages transform into the M2-type macrophage phenotype [42]. In this study, a positive correlation was observed between TRPM7 expression and factor XIIIa^+^ TAM density. The density of Factor XIIIa^+^ TAMs was also high in tumors with high TRPM7 expression. As a result of all these findings, hypothetically, we thought that this positive correlation between TRPM7 expression and Factor XIIIa^+^ TAM density may be responsible for aggressive tumor behaviour and poor prognosis in GC patients with high TRPM7 expression.

In other respects, our study had some limitations. First, the present study was a retrospective study including cases in a single health institution. It therefore may have the potential for selection bias. Second, most of the patients included in the study were at advanced tumor stage, with limitations regarding heterogeneity in the stages of the cases. To solve this problem, CRC cases to be included in the next studies could be divided into two groups as “early stage” or “advanced stage.” Moreover, the study population can be limited to one of these two groups. Third, patients who received chemotherapy in the perioperative period were excluded from the study. However, it is clear that perioperative chemotherapy is important in current treatment modalities of GC. Therefore, it may be appropriate to include patients receiving chemotherapy in the study population in order to investigate whether new and effective potential biomarkers also have predictive and pharmacodynamics value in further studies. Moreover, studies investigating the effects of TRPM7 agonists and antagonists in GC cell lines can be added to clinical studies.

## 5. Conclusion

In this study, it was revealed once again that TNM stage, one of the classical prognostic parameters, is an independent prognostic indicator. High TRPM7 expression is closely related to aggressive tumor behaviour and advanced TNM stage. Moreover, it was clearly demonstrated in multivariate Cox regression analysis that high TRPM7 expression is an independent prognostic indicator for GC. TILs and TAMs, which are important components of TME, have an important effect on GC prognosis. Particularly, high CD8^+^ TIL density has been determined as an independent predictor of a good prognosis. Conversely, the high density of Factor XIIIa^+^ TAMs was significantly correlated with aggressive tumor behavior and poor prognosis. Ultimately, it has been shown that high TRPM7 expression and increased Factor XIIIa^+^ TAM density are closely related to clinically poor prognosis in GC. However, further studies are needed to understand the mechanisms by which this occurs at the molecular level and perhaps for new target treatment options.

## Figures and Tables

**Table 1:** Basic clinicopathological characteristics of the patients and the effects of these parameters on overall survival (univariate analysis).

Parameters	*n* (%)	Mean survival ± SD	Overall survival	
			HR (95% CI)	*p* value
Gender				
Male	126 (61.8%)	37.46 ± 14.23	1.02 (0.75–1.40)	0.862
Female	78 (38.2%)	37.30 ± 13.82		
Age				
≤60 year	74 (36.3%)	37.54 ± 13.52	1.01 (0.73–1.39)	0.939
>60 year	30 (63.7%)	37.32 ± 13.78		
Size				
≤5 cm	79 (38.7%)	40.25 ± 13.53	1.35 (0.98–1.85)	0.060
>5 cm	125 (61.3%)	35.60 ± 14.11		
Site				
Proximal	47 (23.0%)	33.65 ± 13.81	0.43 (0.27–0.69)	<0.001
Middle	63 (30.9%)	37.79 ± 12.34		
Distal	54 (26.5%)	44.55 ± 14.09		
Diffuse	40 (19.6%)	31.52 ± 12.89		
Borrmann				
Type 1	28 (13.7%)	41.10 ± 12.45	1.33 (0.81–2.18)	0.254
Type 2	60 (29.4%)	37.13 ± 14.55		
Type 3	76 (37.3%)	36.64 ± 14.22		
Type 4	40 (19.6%)	36.65 ± 14.07		
Lauren				
Intestinal	155 (76.0%)	38.39 ± 14.04	1.47 (0.98–2.20)	0.057
Diffuse	33 (16.2%)	33.24 ± 13.72		
Indeterminate	16 (7.8%)	36.37 ± 13.94		
Histopathological type				
Adenocarcinoma-NOS	110 (53.9%)	36.60 ± 12.73	0.90 (0.76–1.08)	0.275
Mucinous	50 (24.5%)	38.54 ± 15.19		
Signet ring cell	32 (15.7%)	38.87 ± 15.75		
Poorly cohesive	12 (5.9%)	36.08 ± 16.92		
Histologic grade				
Grade 1	24 (11.8%)	48.20 ± 12.79	2.74 (1.48–5.09)	0.001
Grade 2	124 (60.8%)	36.62 ± 13.16		
Grade 3	56 (27.5%)	34.50 ± 14.54		
Goseki grade				
Grade 1	73 (35.8%)	36.42 ± 11.80	0.95 (0.84–1.07)	0.430
Grade 2	23 (11.3%)	38.52 ± 15.97		
Grade 3	44 (21.6%)	39.40 ± 15.50		
Grade 4	64 (31.4%)	36.73 ± 14.78		
Lymphovascular invasion				
Absent	74 (36.3%)	39.98 ± 15.33	1.45 (1.04–2.00)	0.022
Present	130 (63.7%)	35.80 ± 12.99		
Perineural invasion				
Absent	90 (44.1%)	38.93 ± 13.20	1.12 (0.81–1.54)	0.465
Present	114 (55.9%)	36.53 ± 14.48		
Depth of invasion				
pT1	12 (5.9%)	58.16 ± 4.78	2.62 (2.07–3.32)	<0.001
pT2	24 (11.8%)	52.33 ± 11.19		
pT3	112 (54.9%)	36.43 ± 12.42		
pT4	56 (27.5)	28.48 ± 9.24		
Lymph node metastasis				
Absent	25 (12.3%)	55.84 ± 7.75	2.18 (1.83–2.59)	<0.001
1–2	44 (21.6%)	45.84 ± 12.25		
3–6	62 (30.4%)	34.19 ± 11.01		
≥7	73 (35.8%)	28.72 ± 9.85		
Distant metastasis				
Absent	132 (64.7%)	43.96 ± 12.23	5.65 (4.04–7.89)	<0.001
Present	72 (35.3%)	25.37 ± 7.82		
TNM stage				
I	20 (9.8%)	58.65 ± 3.84	4.09 (3.26–5.12)	<0.001
II	48 (23.5%)	48.45 ± 10.77		
III	76 (37.3%)	35.75 ± 8.51		
IV	60 (29.4%)	23.56 ± 6.23		

SD: standard deviation; HR: hazard ratio; CI: confidence interval.

**Table 2:** Relationship between prognostic parameters and overall survival according to multiple Cox regression analysis.

Prognostic parameters	Overall survival (multivariate)	
	HR (95% CI)	*p* value
Gender (male/female)	1.12 (0.80–1.57)	0.502
Age (<60, ≥60)	1.26 (0.91–1.76)	0.161
Site (proximal/middle/distal/diffuse)	0.95 (0.82–1.11)	0.579
Histological grade (1/2/3)	1.03 (0.78–1.36)	0.797
Lymphovascular invasion (absent/present)	0.98 (0.69–1.41)	0.952
Depth of invasion (pT1/pT2/pT3/pT4)	0.78 (0.58–1.06)	0.120
Lymph node metastasis (absent/1-2/3-6/≥7)	1.19 (0.95–1.50)	0.145
TNM stage (I/II/III/IV)	3.43 (2.47–4.76)	<0.001
CD4^+^ TIL density (low/high)	0.45 (0.15–1.30)	0.142
CD8^+^ TIL density (low/high)	0.22 (0.12–0.39)	<0.001
Factor XIIIa^+^ TAM density (low/high)	1.38 (0.49–3.88)	0.540
TRPM7 expression (low/high)	8.70 (5.20–14.54)	<0.001

HR: hazard ratio; CI: confidence interval.

**Table 3:** Effect of prognostic parameters on recurrence risk according to multivariate Cox regression analysis.

Prognostic parameters	Overall survival (multivariate)	
	HR (95% CI)	*p* value
Gender (male/female)	1.29 (0.93–1.78)	0.119
Age (<60, ≥60)	1.06 (0.78–1.44)	0.696
Site (proximal/middle/distal/diffuse)	0.99 (0.85-1.14)	0.892
Histological grade (1/2/3)	1.28 (1.01-1.64)	0.041
Lymphovascular invasion (absent/present)	1.09 (0.78-1.51)	0.604
Depth of invasion (pT1/pT2/pT3/pT4)	0.81 (0.63-1.05)	0.126
Lymph node metastasis (absent/1-2/3-6/≥7)	1.04 (0.84-1.29)	0.694
TNM stage (I/II/III/IV)	3.07 (2.26-4.18)	<0.001
CD4^+^ TIL density (low/high)	0.55 (0.24-1.26)	0.162
CD8^+^ TIL density (low/high)	0.23 (0.13-0.40)	<0.001
Factor XIIIa^+^ TAM density (low/high)	1.26 (0.57-2.80)	0.557
TRPM7 expression (low/high)	4.57 (2.93-7.12)	<0.001

HR: hazard ratio; CI: confidence interval.

## Data Availability

Present study data is from the “Department of Pathology, Affiliated faculty of medicine, Fırat University, Elazığ (23200), Turkey,” and from patients with gastric cancer who underwent resection for gastric cancer at the Hospital of Medical Faculty of Fırat University, from January 2009 to January 2016. Since the use of raw data is subject to the special permission of the ethics committee of Fırat University, Faculty of Medicine, and the head of scientific research, these data cannot be provided for personal, research, and/or commercial purpose.
